# Application report of automatic unlocking baseplate in radiotherapy

**DOI:** 10.1002/acm2.13778

**Published:** 2022-09-12

**Authors:** Lintao Li, Xianliang Wang, Xin Xin, Ming Fan, Shun Lu, Wei Wang, Gang Yin

**Affiliations:** ^1^ Department of Radiation Oncology, Sichuan Cancer Hospital & Institute, Sichuan Cancer Center, School of Medicine University of Electronic Science and Technology of China Chengdu China; ^2^ Radiation Oncology Key Laboratory of Sichuan Province Chengdu China; ^3^ Klrity Medical&Equipment Co. Ltd. Guangzhou China

**Keywords:** automatic unlocking, baseplate for radiotherapy, head and neck tumor patients, radiotherapy, safety protection device

## Abstract

**Purpose:**

To reduce the potential risk during radiotherapy treatment of patients with head and neck tumors, we improved upon the design of an existing immobilization device by adding a feature to improve patient safety during emergency releases, and we verified its clinical application.

**Method:**

We designed an improved automatic unlocking baseplate (AUB), and conducted a dosimetry comparison with Solo Align Full Body System (SAFBS, Klarity, China). The dosimetry comparison included dose‐attenuation measurements and results from human simulation. We selected four points for measurement to allow comparison between the SAFBS and our AUB. A simulated human body model was used for CT scanning, whereby the target area and structure and simulated radiotherapy plan were conducted according to the American Academy of Pain Medicine Task Group–119 report (TG‐119), whereby the dose differences were compared. The purpose of the clinical test was to verify the reliability of the AUB system in practical clinical applications. The application tests were conducted in CT simulation (CT‐sim) and treatment rooms. The test included assessments of the stability of the system and the reliability of our device.

**Results:**

The dose‐attenuation measurements of the two baseplates were as follows: The transmission values with our unlocking system were 0.10% higher at the first point and 0.67% lower at the third. The same dose was obtained at points 2 and 4. In the simulation study, the PTV of the AUB was lower than that of the SAFBS, including 0.39% lower *D*_99_ and 0.18% lower *D*_90_. Among the organ‐at‐risk doses, the average dose of the AUB in the spinal cord was 0.6% higher than that of the SAFBS, and the average dose in the left and right parotid glands was more than 1.4% lower than that of SAFBS. The clinical test results were applied in treatment room and a CT‐sim room, which show a 100% success rate after being unlocked more than 5000 times.

**Conclusion:**

The AUB designed for head and neck patients had good functional versatility, the dose distribution met the requirements, and the automatic unlocking function was demonstrated to be stable and reliable.

## 1 INTRODUCTION

Head and neck cancers are commonly diagnosed malignant tumors.^1^ Currently, chemotherapy and radiotherapy are the main treatments for head and neck tumors.^2^, ^3^

In the process of radiotherapy, in order to ensure the accuracy of the dose delivery during multiple passes, fixing devices are used to fix the patient. Typically, thermoplastic material is applied to control any positional deviation to a submillimeter level^4^ during radiotherapy, including CT/MRI and PET‐CT, to allow accurate images of the target area to be obtained.^5^

Contrast agents assist in defining and delineating the tumor area in images. Adverse drug reactions (ADRs), including severe allergic reactions, may occur when using contrast agent.^6^ Regarding enhanced CT, the most common allergic reactions of iodinated contrast media are nausea, vomiting, and aspiration,^7^ which can lead to failure to acquire the necessary images.^8^ When ADR occurs, patients are usually advised to turn their head to one side to reduce the risk of a foreign body and aspiration in trachea.^9^ For patients who are receiving radiotherapy for head and neck tumors, the fixation device during CT‐simulation (CT‐sim) can be dangerous during ADRs, as the patient cannot turn their head or open their mouth to vomit or ease their breathing, which increases the risk of aspiration. When ADR occurs, the technician must halt scanning and immediately release the patient from fixation for at least 15 s by entering the scanning room. Cancer patients are relatively older, on average, and they can have serious complications from aspiration, including aspiration pneumonia. Aspiration pneumonia increases the mortality rate of patients with head and neck cancers by 42%.^10^ In addition to ADRs, there are several risk factors that may lead to asphyxia during radiotherapy, including sudden bleeding,^11^ excessive secretions, and so forth.

In order to achieve the required accuracy during the fixation of patients for imaging in head and neck radiotherapy, and to improve the function of patient releasing from fixation in case of emergency, simultaneously without affecting the distribution of radiation dose and the workload of technicians, we have designed a protective device which is suitable for the thermoplastic mask fixation device that is widely used at present. In the case of an emergency, such as an adverse drug reaction, the safety device allows a patient's restraints to be released immediately.

## 2 MATERIALS AND METHODS

This study was approved by the ethics committee of our institution and complied with the Declaration of Helsinki.

### 2.1 Design and unlock function

**FIGURE 1:**
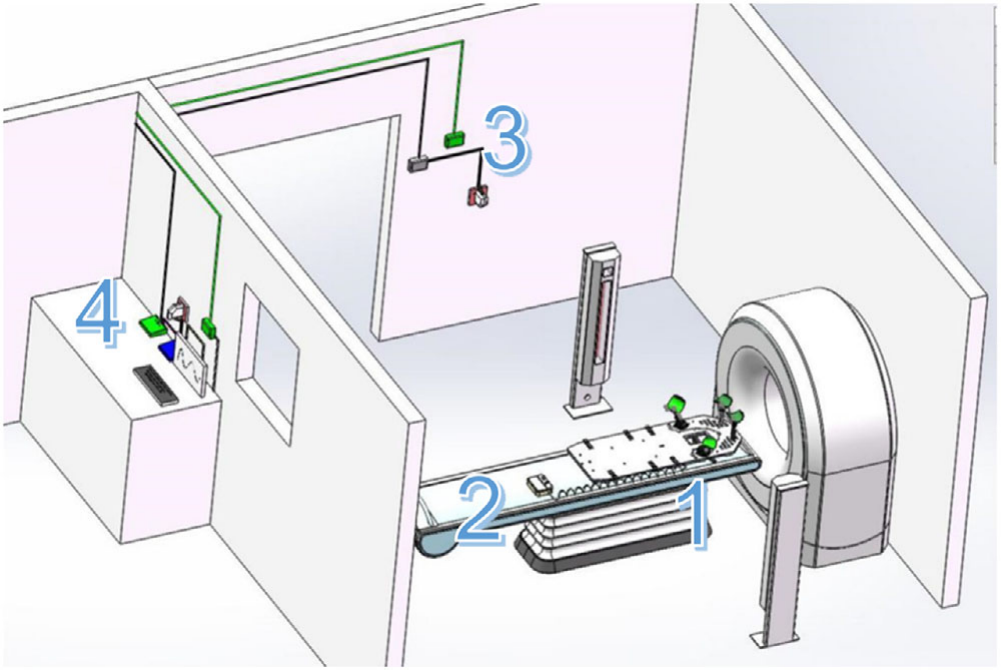
Schematic diagram of the overall design in a typical CT room. The automatic unlocking scheme is divided into four modules: (1) AUB in the CT room; (2) control integrated module in the CT room; (3) Bluetooth connection system; and (4) exterior controller. AUB, automatic unlocking baseplate

The overall diagram of the device is shown in Figure 1. The design of the fixation frame developed here is an improvement based on a preexisting integrated fixation device. Compared to the current U.S. Food and Drug Administrationapproved product, the thickness was increased by 5 mm, and the shape and the size were the same as in the existing product. However, not all materials were high‐density. The electromechanical equipment used was from outside the field. Currently, the typical head, neck, and shoulder thermoplastic mask is fixed by expansion (or locking) nails, and nine holes are used on a fixed plate formed of low‐density material and covered with carbon fiber. Thus, in this design, the success of this function heavily depends onwhetherthe expansion nails can be lifted. An airbag belt was used for inflation, sothat the expansion nails could be pushed out.

**FIGURE 2:**
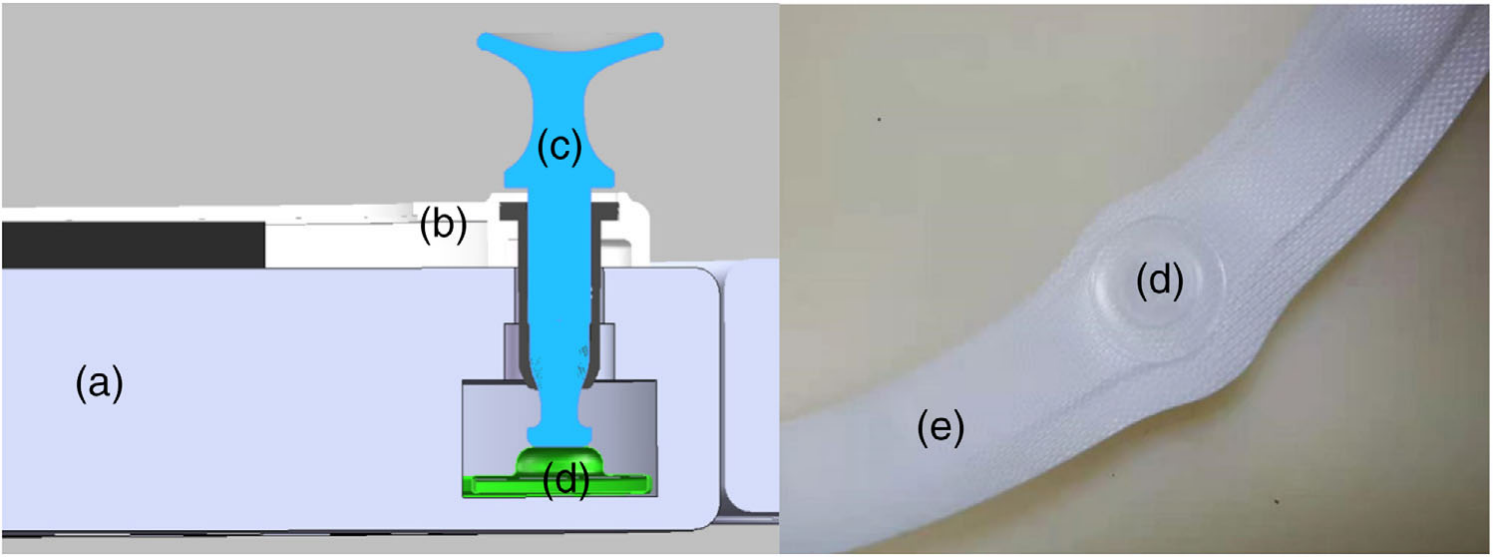
Design of the unlocking function. (a) Base of AUB; (b) thermoplastic outer frame; (c) locking nails used for thermoplastics; (d) airbag contact; and (e) airbag belt. When unlocking, the airbag belt (e) is inflated, and the airbag contact (d) protrudes upward to lift the expansion (or locking) nail (c). AUB, automatically unlocking belt

First, the mold was created, and then the carbon fiber shell was created according to the carbon fiber production process, and a low‐density foam material was wrapped around it. The inflatable belt was placed inside the carbon fiber shell. The unlocking design is shown in Figure 2.

The control box integrated the power supply, air pump, Bluetooth module, network Input/Output module, air valve, and other devices. The type of air pump was G7BL128514‐2; the maximum power was 20 W. The inflation rate was 250 mL/s, and the inflation time was set to 4 s, the inflation volume was 1 L, and the opening diameter was 4 mm. The above functional modules were powered by a rechargeable lithium battery with a power of 6800 mA, and the Bluetooth module was connected to the transmitter that was wired into the control room.

**FIGURE 3:**
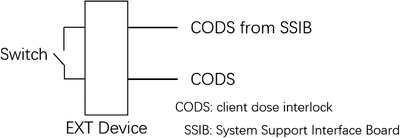
Linear electron accelerator(LINAC) interlock to achieve access; CODS refers to client dose interlock

The control room was equipped with an unlock switch, which was connected to the Bluetooth transmitter in the treatment room via a wired connection. The host device called the protocol stack function “sd_ble_gap_connect ()” to automatically connect the target slave device, which was automatically initialized during start‐up. In the treatment room, we assessed and mitigated the risk of a patient colliding with the frame upon release, and the interlocking function of the accelerator was activated via the user interface of the accelerator. The design diagram is shown in Figure 3. The interlocking start switch was in the control module, and the switch trigger initiated the interlocking function via Bluetooth.

### 2.2 Dose‐attenuation measurement

We used a digital electrometer (Unidos E, PTW) and a finger‐type power chamber (30013, PTW) for isocenter dose test of standard solid water. The dimensions of standard solid water were 30 cm × 30 cm × 1 cm, which could reach 10 cm through multi‐layer accumulation. The laser light corresponds to the ionization chamber at the center of the radiation field, and then, we placed two types of baseplates on top for dose‐attenuation testing.

We used the Elekta Synergy linear accelerator, 6 MV, with a radiation field of 10 cm × 10 cm. The test environment temperature was 24°C, the beam dose was 100 MU, the gantry angle was 0°, and the source to surface distance was set to 100 cm. For our calculations, the dose without the baseplate is D0, the dose‐attenuation value with baseplate is Da, and the formula for the dose‐attenuation rate (F) is as follows:

$$F\;=\frac{D0-Da}{D0}\;\times 100\%$$

**FIGURE 4:**
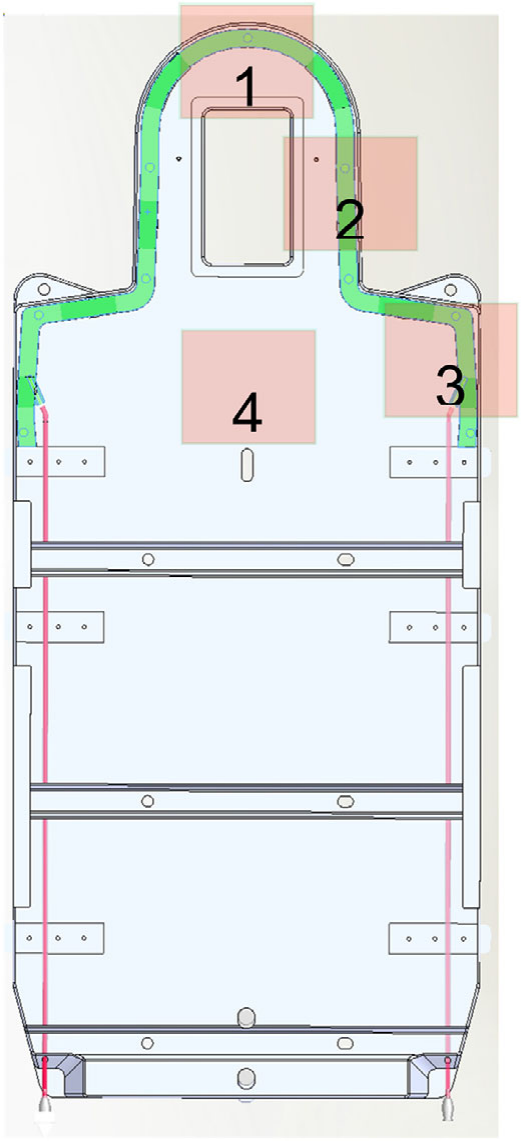
The front view of the AUB in the schematic diagram of the dose‐attenuation test. The green areas represent the airway. The numbers in the figure indicate the dose‐attenuation test field. AUB, automatic unlocking baseplate

Due to the consistency of the baseplate material, the area where the airway would be affected was considered when selecting the test points. Figure 4 shows the specific test points used.

### 2.3 Dosage verification

In this study, computed tomography simulation (CT‐sim, Philips; Brilliance Big Bore, Netherlands), an irradiated simulation model of the human body (China radiation anthropomorphic phantom and mathematical models of human body organs; CDP‐1C, Chengdu FangTuo), thermoplastic mask for head and neck (S‐type masks, Klarity, Guangzhou China), Solo Align Full Body System (SAFBS) and automatic unlocking baseplate (AUB), a digital electrometer (UNIDOS E, PTW), and a finger‐type waterproof ionization chamber (30013, PTW) were used. Head, neck, and shoulder thermoplastic mask was used to secure the irradiated, simulated human body model, and the head and neck scanning sequence were used to collect images in CT‐sim. The two different baseplates used the same center alignment and scanning conditions. The CT parameters were selected as 120 kV, the slice thickness and the slice spacing were 3 mm, and the FOV was 320 mm. We determined the target area and at‐risk organs according to American Academy of Pain Medicine (AAPM) TG‐119,^12^ including PTV, left and right parotid glands, and spinal cord, and we selected the same CT‐sim ball bearings (BBs) point. The CT‐sim image was captured and sent to the Eclipse planning system to design the same plan in accordance with the prescription dose according to the AAPM TG‐119 report.

### 2.4 Clinical test

Clinical testing was conducted in the Radiotherapy Center of the Sichuan Cancer Hospital from June 2021 to November 2021, (No: IIT2021047). The CT‐sim and application in the treatment room were conducted simultaneously and comprised three considerations: (1) verifying the stability of the dose distribution; (2) assessing the reliability of the automatic unlock function during daily use; and (3) the emergency state results as an indicator of the effectiveness of our unlocking device.

## 3 RESULT

### 3.1 The dose‐attenuation test results

The basic value *D0* was 1.050 Gy; we selected four points according to the SAFBS and AUB as previously indicated, and these were measured three times at the same position. The values were averaged, and the results are shown in Table 1.

**TABLE 1:** The dose‐attenuation test results

**Area**	**SAFBS (Gy)**	**Dose‐attenuation rate (F)**	**AUB (Gy)**	**Dose‐attenuation rate (F)**
1	1.035	98.57%	1.037	98.76%
2	1.034	98.48%	1.034	98.48%
3	1.011	96.29%	1.004	95.62%
4	1.038	98.86%	1.038	98.86%

High stability of dose‐attenuation test with deviation less than 0.001 Gy.

Dose‐attenuation test showed a high degree of agreement between the two baseplates, with only significant differences in area 3.

### 3.2 The simulation plan results

**FIGURE 5:**
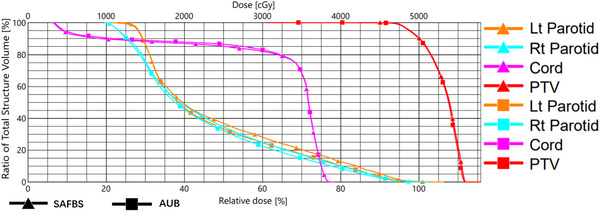
DVH of two simulation plans. The graph is output by the Eclipse planning system and the results show a high degree of consistency.

The DVH plots obtained for the two baseplates are shown in Figure 5.

The results of the simulation plan were like those of the dose‐attenuation test, showing a high degree of interoperability of the two baseplates, and the results are shown in Table 2.

**TABLE 2:** AAPM TG‐119 dosimetry control results

	**PTV**		**Cord**		**Left parotid**		**Right parotid**	
	***D*_99_**	***D*_90_**	**Max**	**Mean**	***V*_30‐_**	**Mean**	***V*_30_**	**Mean**
SAFBS	4747.59	5009.56	77.4%	63.5%	2896.88	48.8%	2723.35	46.3%
AUB	4729.05	5000.6	78.4%	64.1%	2659.48	47.1%	2600.23	44.9%

Dosage units in the table are cGy; the percentage value is a percentage of the maximum dose limit.

### 3.3 Clinical test

**FIGURE 6:**
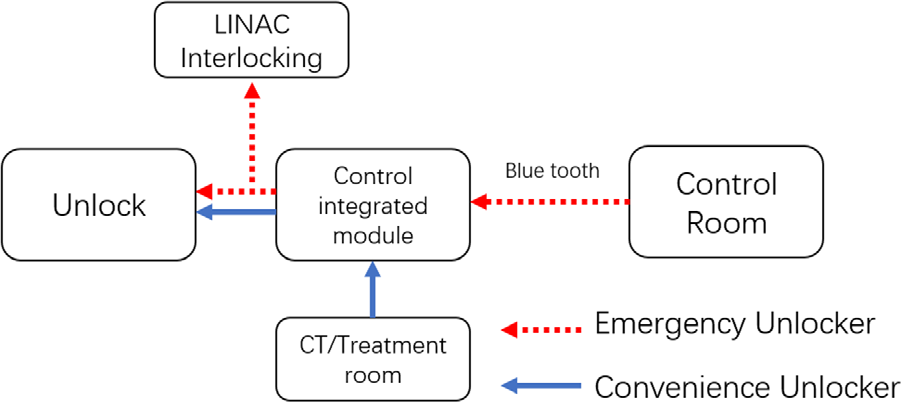
The manual AUB, the diagram shows the use process under different conditions. AUB, automatically unlocking baseplate

After the dosage and functional testing, the clinical trial was launched in the Radiotherapy Center of the Sichuan Cancer Hospital in June 2021, and the clinical trial was conducted in a CT‐sim computer room and a head and neck tumor‐dedicated accelerator computer room. The clinical trial endpoint was 1000 times. In more than 5000 actual applications (i.e., unlocking for daily use), the unlocking success rate was 100% in cases of emergency unlocking. No adverse events were observed during clinical testing. We determined that the recommended use of AUB could be divided into convenience unlocking and emergency unlocking. Convenience unlocking is referring to unlocking for the convenience of medical staff, so they can quickly release the mask. This function was typically used by the therapist in the control room. The process is shown in Figure 6.

## 4 DISCUSSION

For patients receiving radiotherapy, risk factors typically include the risk of the specific treatment and its additional side effects,^13^ the environmental risks in medical facilities, and the risks caused by the disease itself. A retrospective study in the United States showed that 5% of all the radiotherapy patients had been involved in dangerous situations or safety accidents,^14^ and many risk factors were unexpected or undetectable. Head and neck tumors are currently treated using adaptive radiotherapy.^15^ This recommendation increases the proportion of patients undergoing CT‐sim/MR‐sim, and the risk increases accordingly.

During an MR‐sim, the patient wears a fixation device for 20–30 min, however, the patient may need to be ventilated due to difficulties such as claustrophobia or nervousness.^16^ The current application of MR‐guided radiotherapy provides better soft tissue resolution and monitoring during treatment, but the treatment time is up to 40 min, during which patients are exposed to the loud volume of the machine.^17^ As a result, it is more likely that patients will require immobilization devices.

We considered the typical environment of CT and MRI scans when designing the unlocking device, and the current sample was suitable for CT and treatment rooms, but all modules could be manufactured to meet the conditions of MRI scans, which would expand the protection scope of the device. In future research, we will develop an MRI‐adapted unlocking device that meets the requirements for use of MRI‐sim and MRI‐LINAC, such as Unity.

**FIGURE 7:**
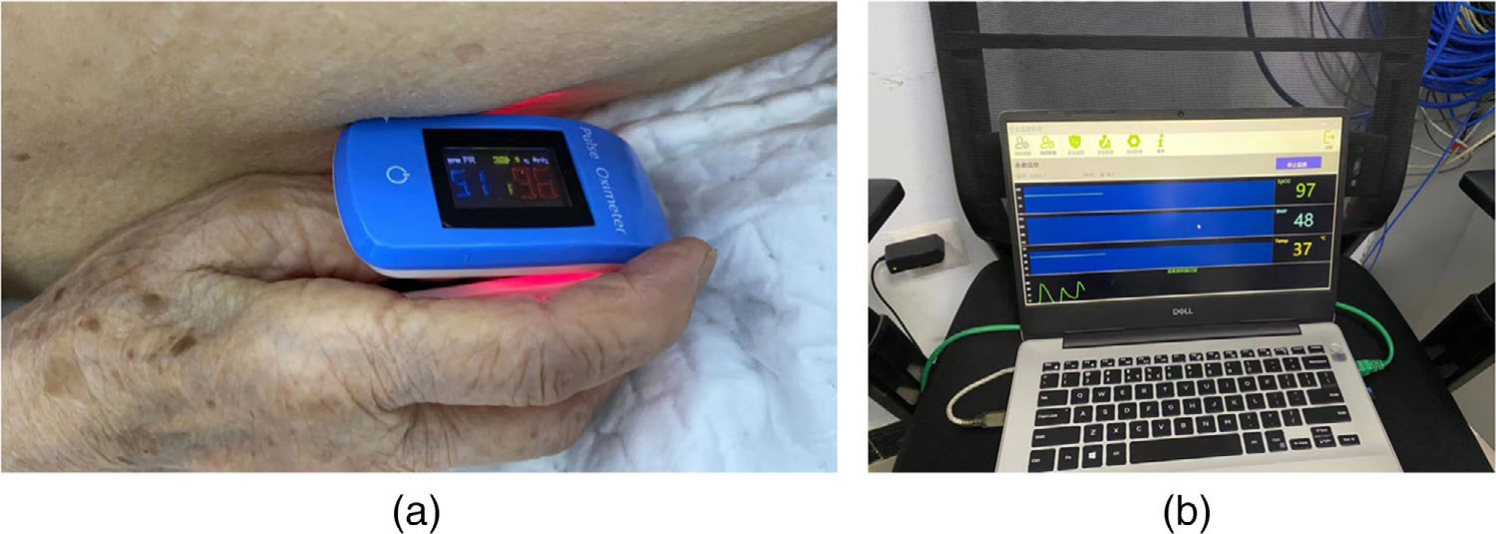
Vital signs monitoring equipment during radiotherapy. (a) The blood oxygen saturation and pulse rate monitor on the patient's fingertip; and (b) the data display interface in the control room

Currently, many facilities use hand‐held alarms to allow the patient to inform the therapist of discomfort. However, if a patient is unable to use the alarm for any reason, such as extreme anxiety or going into shock. fingertip oxygen saturation and pulse rate monitoring devices (Figure 7) transmit relevant data so that the staff in the control room can observe the patient's vital signs. As the stability of the product and the system had not yet been established, we did not link the fluctuation of data to the AUB. We set parameters for changes in blood oxygen and pulse rate; if they fluctuated by more than 20%, the device emits an alarm. In addition, emergencies are typically determined using in‐room cameras to monitor patients, but this method is not always reliable.

In the experiment, in addition to improving patient safety, the therapists involved noted that it improved the efficiency of releasing patients based on its single‐key locking mechanism, which would be of value for radiation therapy centers with a large number of patients.

In this study, we only conducted dose comparison tests for the target areas and at‐risk organs specified in the AAPM TG‐119 report, and we did not compare doses in clinical applications. While radiation therapy appears to benefit patients with head and neck tumors, this has not been established for tumors located elsewhere in the body. As this safety device is multifunctional and modular, it may be useful for additional applications in the future that require patients to be secured in one position for long periods.

## 5 CONCLUSION

In this study, we designed a remote automatic unlocking device that was demonstrated to be highly reliable. It met the radiation penetrability of the dose distribution in the test and did not require changes in the existing fixation method used for radiotherapy patients. The success rate was high and met the emergency release requirements for patients during radiotherapy, and it does not require any changes to the current associated dose delivery methods.

## AUTHOR CONTRIBUTIONS

Lintao Li and Shun Lu made substantial contributions to conception and design. Lintao Li, Ming Fan, Gang Yin, and Xianliang Wang involved in drafting the manuscript or revising it critically for important intellectual content. Lintao Li, Xin Xin, Ming Fan, Wei Wang and Xianliang Wang contributed to acquisition of data, analysis, and interpretation of data. All authors read and approved the final manuscript.
